# Supplementary material for: Visualization of supercritical water pseudo-boiling at Widom line crossover
Source: Nat Commun. 2019 Sep 17;10:4114. doi: 10.1038/s41467-019-12117-5 (PMC6748934; doi:10.1038/s41467-019-12117-5)
Supplement: Supplementary file 1 — Supplementary Information [file 41467_2019_12117_MOESM1_ESM.pdf]

Visualization of supercritical water *pseudo*-boiling at Widom line crossover

Maxim et al.

Supplementary Discussion

Details about the monolith.

The porous material referred as monolith has an apparent volume in the reactor of 2.15 cm^3 (Supplementary Figure 1a) and weighs about 0.48 g (density of 0.225 g cm^{-3}). The monolith is made of activated carbon fibers with average diameter around 17 µm, as determined by SEM images analysis (Supplementary Figs. 1b, d). The internal surface area measured from N_2 adsorption at 77 K is 1360 m^2 g^{-1}, calculated by the Brunauer – Emmett – Teller (BET) method. The total pore volume available for N_2 condensation (width w < 300 nm) is 0.62 cm^3 g^{-1}, of which 0.57 cm^3 g^{-1} is in pores with width w < 35 nm and 0.53 cm^3 g^{-1} is in pores w < 2 nm (micropores). Further characterization from combined data of N_2 adsorption (77 K) and CO_2 adsorption (273 K) showed that micropores have bimodal pore distribution, with maxima at 0.5-0.6 nm (ultramicropore) and 1.0-1.2 nm (supermicropores, as defined by IUPAC). See Supplementary Figure 2.

Using the above information, it follows that the fibers in the particular monolith used in our experiments expose an external surface of about 0.01 m^2 while the internal surface in the micropores (w < 2 nm) is a gigantic 657 m^2. Similarly, the apparent volume of all carbon fibers in the monolith is only 0.47 cm^3, of which more than half (0.26 cm^3) is contained in internal pores. In contrast, the apparent volume of the monolith itself is 2.15 cm^3. The open interfibrillar volume in the monolith is 1.67 cm^3 (78 % of the apparent monolith volume). The numbers tell us that we cannot neglect the huge area and volume of internal pores (micropores) in the monolith.

However, further water adsorption experiments and the analysis of the micropore-filling mechanism (see below) revealed that adsorption forces for water adsorbed in the micropores are so strong that this confined water only desorbed after the *pseudo*-boiling was initiated in the interfibrillar space.

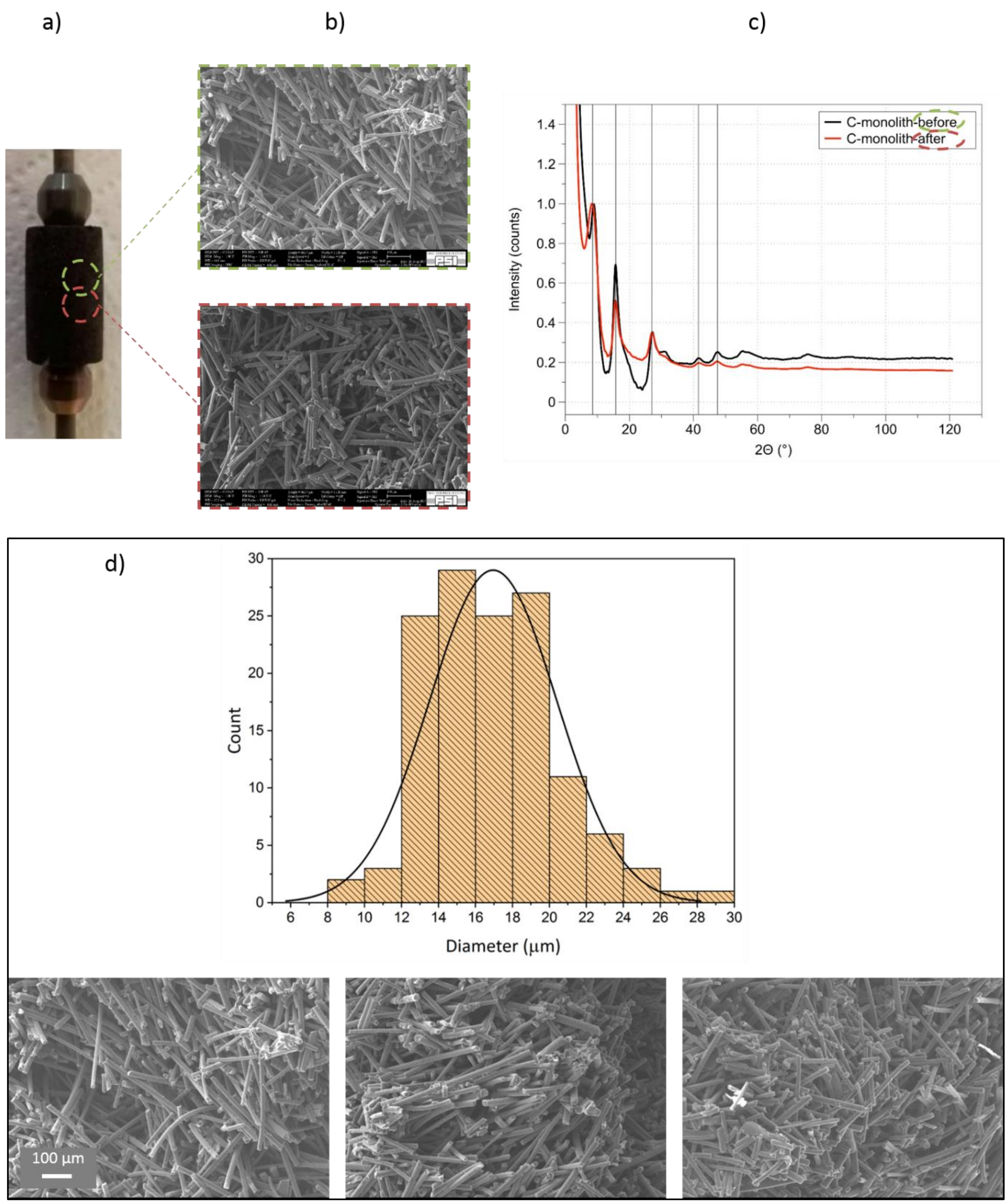

Supplementary Figure 1 a) Image of the carbon monolith sample fixed on the thermocouple support; b) SEM images of the monolith before (up) and after (down) and c) HR-XRD patterns for the monolith samples before (black line) and after (red line) the experiment under supercritical water conditions; d) Histogram of monolith's fibers diameter determined based on measurements from the SEM images. Source data for the XRD pattern and for the histogram are provided as a Source Data file.

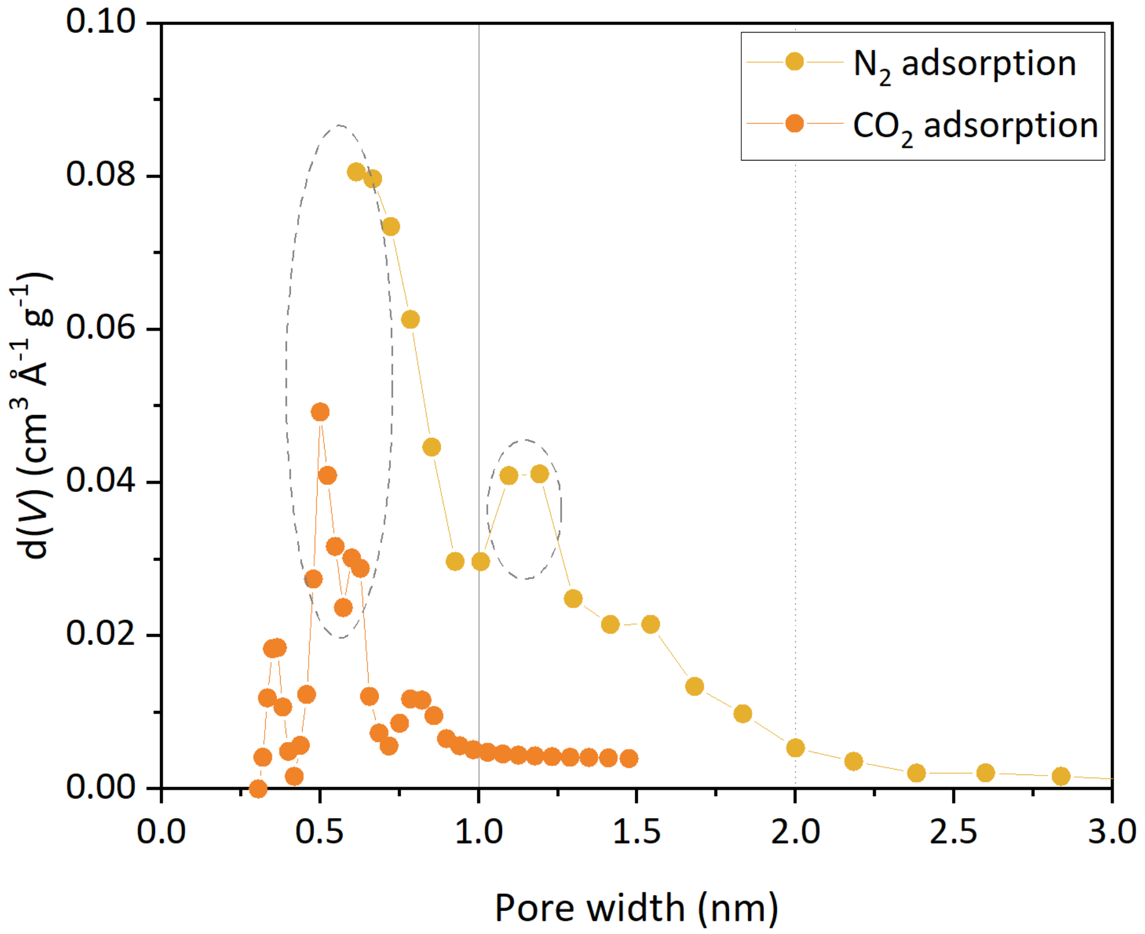

Supplementary Figure 2 Monolith's micropore size distribution determined by both N_2 adsorption and CO_2 adsorption methodsThe plot of incremental pore volume *vs* pore width shows that both methods agree on the position of dominant pores; grey circles indicate the bimodal size distribution: ultramicropores (width <0.7 nm) and supermicropores (width >0.7 nm). Source data are provided as a Source Data file.

Setup used for *in-situ* neutron imaging

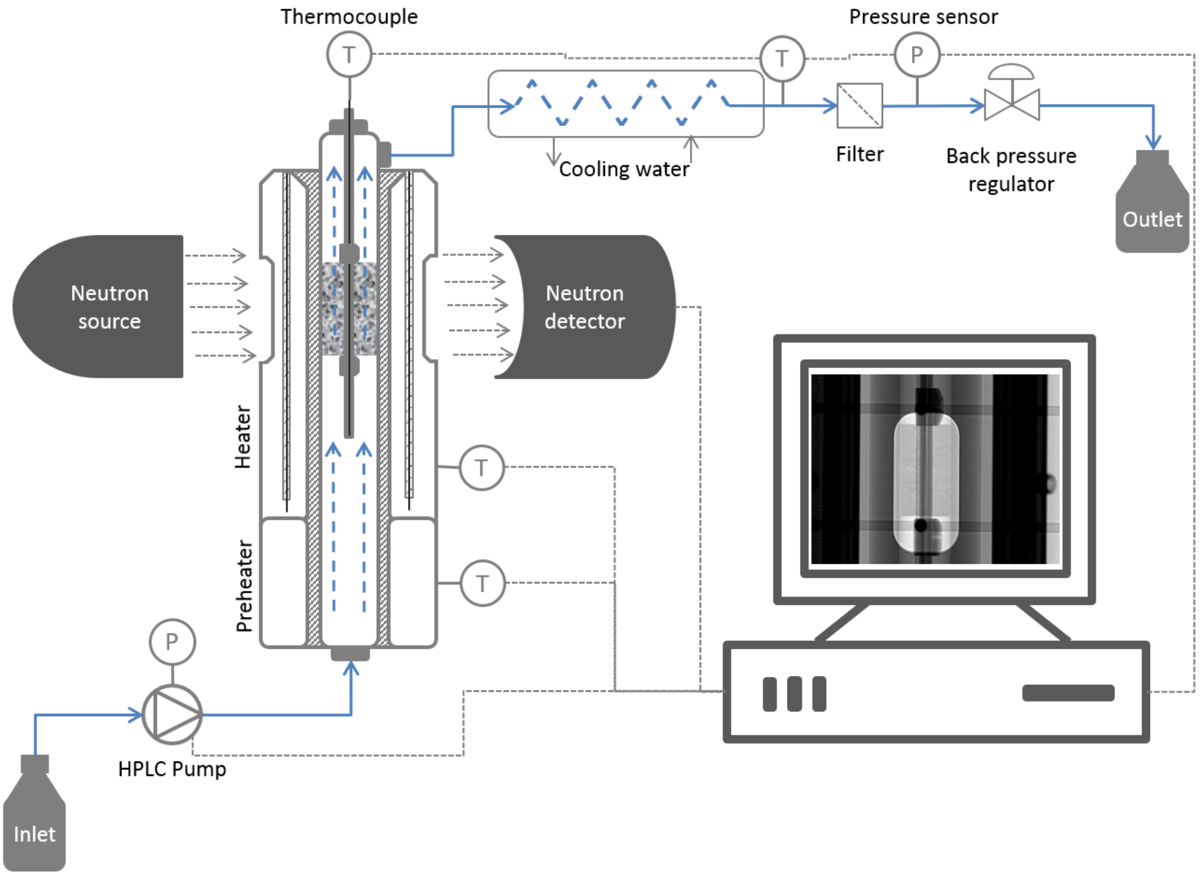

Supplementary Figure 3 Schematic representation of the setup used for *in-situ* neutron imaging under supercritical water conditions; the main components are: the continuous flow tubular reactor, high-precision liquid chromatography pump, preheater, aluminum block heater and back pressure regulator; the direction of water flow is indicated by blue arrows; the temperature, the pressure and the water flow rate is controlled and monitored on-line and the values are recorded every 10 s

Analysis of water density in activated carbon fiber micropores

In absence of direct measurements of adsorbed amounts in supercritical conditions we did dynamic vapor sorption measurements of water adsorption at 25, 35 and 55 °C on an activated carbon fiber with similar physical properties (BET surface area, pore volume and pore size distribution) as the fibers used for fabrication of the monolith. As detailed in the Methods section, the experimental data were fitted by a unique characteristic adsorption curve expressed by the Dubinin-Astakhov (DA) equation for the micropore filling process (Equation 2 in Methods). Supplementary Figure 4 shows the characteristic DA adsorption curve with all experimental data on the same isotherm. The inset in the same figure shows the actual adsorption and desorption measurements with water on activated carbon fibers.

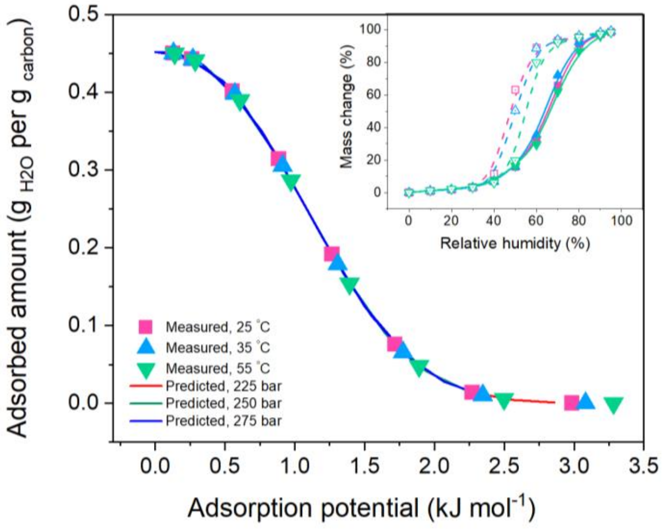

Supplementary Figure 4: Characteristic adsorption curve for activated carbon fibers showing experimental data measured in the lab at subcritical conditions and the predicted behavior for supercritical conditions. The inset shows adsorption and desorption isotherms measured in the lab at three different temperatures (298, 308 and 328 K). Source data are provided as a Source Data file.

Since the DA characteristic parameters E and n are quite insensitive to temperature,[1] it is possible to use the DA equation over a broad range of temperatures and pressures, in subcritical conditions. Extending it to supercritical conditions was first proposed by Dubinin, who replaced the subcritical saturation pressure P_0 with a hypothetical *pseudo*-saturation pressure of supercritical gas (P_s). According to Dubinin and his collaborators, each adsorbent-adsorbate system follows a unique characteristic curve which describes adsorbed amounts (W) dependence on the adsorption potential A over a broad range of conditions, both sub- and supercritical.[2] This approach was further developed into complex analytical methods for evaluation of adsorption in supercritical conditions using the DA equation.[3-5] However, for the limited purpose of this discussion we used the simpler definition proposed by Dubinin for the *pseudo*-saturation pressure:

$$P_{\mathrm{s}} = \left(\frac{T}{T_{\mathrm{CP}}}\right)^2 P_{\mathrm{CP}} \qquad \text{Supplementary Equation 1}$$

where the critical point conditions for water are P_{CP} = 221 bar and T_{CP} = 647 K. We can now use the DA equation to estimate the amounts adsorbed in micropores at supercritical conditions. We used P_{pb} instead of the Dubinin's *pseudo*-saturation pressure and the constant pressure of each experiment (P_{exp}) for the current pressure. Thus, the adsorption potential for subcritical conditions

$$A_s = RT\ln(P_0/P) \quad \text{where } P_0 > P \qquad \text{Supplementary Equation 2}$$

was modified for supercritical conditions as:

$$A_s = RT\ln\left(P_{pb}/P_{exp}\right) \quad \text{where } P_{pb} > P_{exp} \qquad \text{Supplementary Equation 3}$$

Here P_{exp} is the constant pressure of each experiment and P_{pb} is the *pseudo*-boiling pressure calculated for supercritical conditions. The latter depends on temperature according to the generalized equation proposed by Banuti [6] :

$$P_r^* = e^{\left[\frac{B}{\min(T_r^*,1)}(T_r^*-1)\right]} \qquad \text{Supplementary Equation 4}$$

where P_r^* = P_{pb}/P_{CP} and T_r^* = T_{pb}/T_{CP} are the reduced pressures and temperatures on the *pseudo*-boiling line and B is the characteristic constant for water (B = 6.479) from Table 1 of Banuti's paper [6]. The plot of Supplementary Equation 4 shown in Supplementary Figure 5 is linear at $T_r < 1$ and has a slightly higher slope at $T_r > 1$.

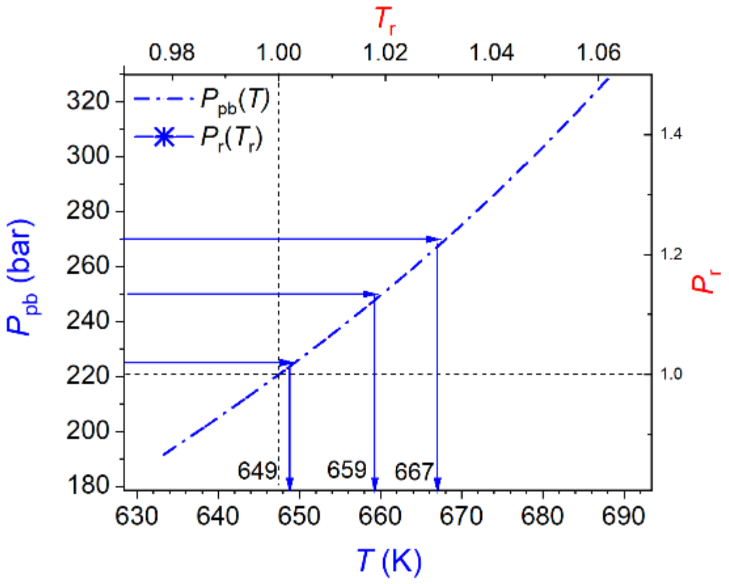

Supplementary Figure 5: Relationship between reduced pressure and reduced temperature P_r=$P_r(T_r)$ below and above the critical point. This is equivalent with Banuti's *pseudo*-boiling line, P_{pb}=$P_{pb}(T)$. Actually, the two lines are undistinguished in this figure. When represented in absolute values, the arrows show the estimated *pseudo*-boiling temperature for each of the three experimental pressures. Compare with Fig. 1b in the manuscript. Source data are provided as a Source Data file.

The reduced pressures of our three experiments are marked by arrows, and the corresponding *pseudo*-boiling temperatures match the experimental temperatures (within the experimental error of ± 1.5 K) – see Table 1 in the manuscript.

The micropore densities can now be calculated in supercritical conditions using the DA isotherm, Equation 2 with the subcritical adsorption potential Supplementary Equation 2 replaced by the supercritical adsorption potential Supplementary Equation 3 and with the pressure vs. temperature relationship on the *pseudo*-boiling line from Supplementary Equation 4. The results were plotted versus temperature in Supplementary Figure 6 for each constant experimental pressure, P_{exp}. Before the *pseudo*-boiling temperature ($T < T_{pb}$) the interfibrillar voids are filled by liquid water, and water in the micropores is still packed to the maximum local density (0.86 g cm^{-3}) imposed by the strong field of confining forces. As the temperature increases ($T > T_{pb}$) and water in the interfibrillar volume changes to GL at the *pseudo*-boiling temperature, the equilibrium with water in the micropores is described by the equivalent of DA equation for supercritical conditions:

$$W = W_0\left[-\left(\frac{RT\ln(P_{pb}/P_{exp})}{E}\right)^n\right] \qquad \text{Supplementary Equation 5}$$

where $P_{exp} < P_{pb}$. At each P_{exp} the amount in micropores (W) decreases, first slower and then faster, as the temperature increases further. The higher the P_{exp} the higher is the T_{pb} where desorption begins. Before the final temperature of our experiments the amount of water adsorbed in micropores drops to about one third at P_{exp} = 225 bar, but it is still very close to its maximum at P_{exp} = 270 bar.

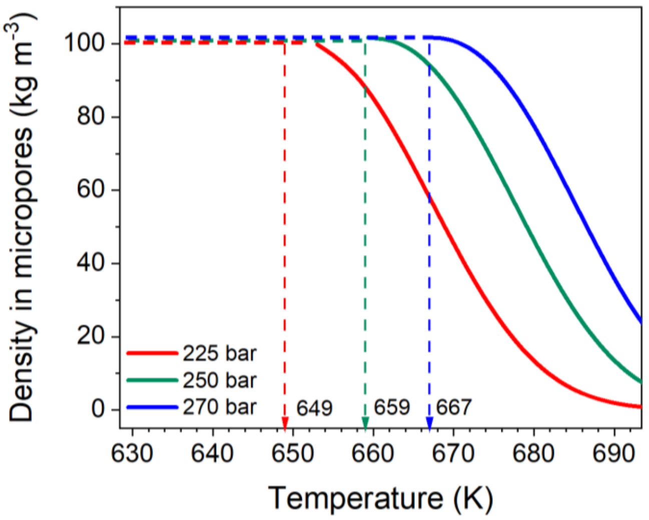

Supplementary Figure 6 Density of water adsorbed in micropores (normalized to monolith's volume) at the three constant pressures of the experiment. With the increase in temperature in supercritical conditions, desorption from micropores takes place within approximately 30 K from the respective T_{pb} at each pressure (marked in figure). Within the range of experimental temperatures, significant desorption was observable only at 225 bar (and partially at 250 bar) below the maximum temperature (673 K) of experiments. Source data are provided as a Source Data file.

In conclusion, the amount of water in the micropores corresponds to the maximum micropore capacity up to the *pseudo*-boiling temperature (T_{pb}). The latter depends on the experiment's pressure (P_{exp}) through Supplementary Equation 5. When the maximum micropore capacity (W_0 = 0.45 g/g) is converted to volumetric density and is further normalized to the monolith's volume (2.15 cm^3), the highest density contribution of micropore-confined water is only 0.1 g cm^{-3} = 100 kg m^{-3} (below the *pseudo*-boiling temperature). This is much lower than the LL density of pressurized water (about 700 kg m^{-3}) and half of its GL density (about 200 kg m^{-3}). After *pseudo*-boiling, the contribution of micropores confined water drops continuously with the increase of temperature.

Therefore, this analysis supports the conclusion that the large variations of water density revealed by neutron imaging are not caused by major density changes in micropore-confined water. The density changes revealed by neutron imaging occur in the interfibrillar space, which represents close to 80 % of monolith's volume. Micropores play a minor role in the observed density variations, and only above the *pseudo*-boiling temperature. The volume where LL and GL phases coexist during conversion from LL to GL is the large interfibrillar space in the monolith. Further confirmation that water confined in micropores does not play effectively a role in LL to GL conversion could be obtained from experiments with monolith made from non-porous carbon fibers or non-porous graphite foam.

Supplementary References

1 Thommes, M., Morlay, C., Ahmad, R. & Joly, J. P. Assessing surface chemistry and pore structure of active carbons by a combination of physisorption (H_2O, Ar, N_2, CO_2), XPS and TPD-MS. *Adsorption-Journal of the International Adsorption Society* **17**, 653-661 (2011).

2 Dubinin, M. M. The potential theory of adsorption of gases and vapors for adsorbents with energetically nonuniform surfaces. *Chemical Reviews* **60**, 235-241 (1960).

3 Srinivasan, K., Saha, B. B., Ng, K. C., Dutta, P. & Prasad, M. A method for the calculation of the adsorbed phase volume and pseudo-saturation pressure from adsorption isotherm data on activated carbon. *Physical Chemistry Chemical Physics* **13**, 12559-12570 (2011).

4 Kaneko, K. & Murata, K. An analytical method of micropore filling of a supercritical gas. *Adsorption-Journal of the International Adsorption Society* **3**, 197-208 (1997).

5 Amankwah, K. A. G. & Schwarz, J. A. A modified approach for estimating pseudo-vapor pressures in the application of the Dubinin-Astakhov equation. *Carbon* **33**, 1313-1319 (1995).

6 Banuti, D. T., Raju, M. & Ihme, M. Similarity law for Widom lines and coexistence lines. *Physical Review E* **95**, 052120 (2017).
