# Supplementary material for: Visualization of supercritical water pseudo-boiling at Widom line crossover
Source: Nat Commun. 2019 Sep 17;10:4114. doi: 10.1038/s41467-019-12117-5 (PMC6748934; doi:10.1038/s41467-019-12117-5)
Supplement: Supplementary file 3 — Description of Additional Supplementary Files [file 41467_2019_12117_MOESM3_ESM.docx]

**Description of Additional Supplementary Files**

**File Name: Supplementary Movie 1**

**Description:** Water system crossing Widom line at 225 bar

**File Name: Supplementary Movie 2**

**Description:** Water system crossing Widom line at 250 bar

**File Name: Supplementary Movie 3**

**Description:** Water system crossing Widom line at 270 bar
